# Radiological Evaluation of Mandibular Residual Ridge Resorption and Its Association With Systemic Biochemical Markers in Completely Edentulous Participants: A Clinical Study

**DOI:** 10.7759/cureus.114516

**Published:** 2026-08-14

**Authors:** Chandrika P Mishra, Tapan K Patro, Angurbala Dhal, Lokanath Garhnayak, Ullash Kumar, Saffalya Nayak, Fakir Mohan Debta

**Affiliations:** 1 Prosthodontics and Crown &amp; Bridge, Srirama Chandra Bhanja Dental College &amp; Hospital, Cuttack, IND; 2 Biochemistry, Srirama Chandra Bhanja Dental College &amp; Hospital, Cuttack, IND; 3 Oral Medicine and Radiology, Srirama Chandra Bhanja Dental College &amp; Hospital, Cuttack, IND

**Keywords:** alkaline phosphatase, biochemical markers, calcium, complete edentulism, phosphorus, residual ridge resorption, vitamin d, vitamin k, wical and swoope method

## Abstract

Background

Residual ridge resorption (RRR) is a chronic, progressive, and multifactorial process that occurs following tooth loss, resulting in continuous alveolar bone loss and potentially compromising prosthodontic rehabilitation. Systemic biochemical factors involved in bone metabolism may influence the extent of mandibular ridge resorption in completely edentulous individuals.

Methodology

This study included 54 completely edentulous participants aged 45-80 years. Mandibular RRR was assessed using digital panoramic radiographs based on the Wical and Swoope method. Serum levels of vitamin K, vitamin D, ionized calcium, phosphorus, and alkaline phosphatase (ALP) were estimated using standardized laboratory techniques. Spearman correlation analysis, the Mann-Whitney U test, and stepwise linear regression were performed to evaluate the association between biochemical parameters and RRR.

Results

RRR showed a significant negative correlation with serum vitamin K (r=−0.42, p=0.002) and serum phosphorus (r=−0.32, p=0.02), and a significant positive correlation with serum calcium (r=0.38, p=0.005). No significant associations were observed with serum vitamin D (p=0.08) or ALP (p=0.49). Stepwise linear regression analysis identified vitamin K as a significant predictor of ridge resorption in the overall study population (R²=0.142, p=0.003) and among males (R²=0.096, p=0.04), whereas serum calcium emerged as a significant predictor among females (R²=0.253, p=0.014). No significant gender-based differences were observed in ridge resorption or biochemical parameters.

Conclusions

Lower serum vitamin K and phosphorus levels were associated with increased mandibular RRR, while higher serum calcium levels were associated with greater bone loss. Vitamin K emerged as a significant predictor of RRR, highlighting its potential role in maintaining alveolar bone integrity. These findings suggest that systemic biochemical markers may influence to mandibular bone changes in completely edentulous individuals and could aid in identifying patients at risk for accelerated ridge resorption.

## Introduction

Complete edentulism is commonly associated with residual ridge resorption (RRR), a chronic and irreversible loss of alveolar bone following tooth extraction that compromises denture support, stability, and retention. The mandible is particularly susceptible to greater resorption than the maxilla due to anatomical and biomechanical differences. RRR is a multifactorial process influenced by local and systemic factors, including functional loading, denture-associated determinants, age, hormonal status, nutrition, and bone metabolism, resulting in considerable interindividual variability [1-3]. Precise assessment of RRR is essential for diagnosis, treatment planning, and long-term prosthodontic outcomes. Panoramic radiography is widely used for this purpose because it provides comprehensive visualization of mandibular bone morphology with minimal radiation exposure [4,5]. Wical and Swoope proposed that the bone inferior to the mental foramen represents approximately one-third of the original mandibular height, enabling estimation of pre-resorptive mandibular dimensions in edentulous patients [4]. Expressing bone loss as a proportion of the original mandibular height minimizes the effects of anatomical and radiographic variations [4,5]. Because RRR reflects continuous bone remodelling, systemic biochemical markers such as involved in skeletal metabolism may influence its progression.

Vitamin K plays a vital role in bone metabolism by promoting osteocalcin γ-carboxylation, which facilitates calcium binding and bone mineralization. Working synergistically with vitamin D and calcium, it helps maintain bone integrity and may reduce RRR; therefore, its deficiency could contribute to alveolar bone loss in edentulous individuals [6]. Vitamin D is essential for calcium-phosphorus homeostasis and bone mineralization by enhancing the intestinal absorption of these minerals. In elderly individuals, vitamin D deficiency is common because of reduced dietary intake, limited sunlight exposure, and decreased cutaneous synthesis. Calcium is essential for bone strength, and its homeostasis is tightly regulated by hormonal mechanisms. When calcium intake or absorption is inadequate, the body compensates by mobilizing calcium from the skeleton, resulting in increased bone resorption. This process may contribute to accelerated mandibular RRR in edentulous individuals [7].

Alkaline phosphatase (ALP), particularly bone-specific ALP (BALP), is an important marker of osteoblastic activity and bone formation. It facilitates osteoid mineralization by increasing local phosphate availability for mineral deposition. Altered ALP levels may reflect changes in bone remodelling, making serum ALP a useful indicator of bone metabolic activity and its potential association with mandibular RRR in edentulous individuals [8].

Phosphorus, primarily in the form of inorganic phosphate, is essential for bone mineralization and hydroxyapatite formation. Its serum levels are tightly regulated by parathyroid hormone, vitamin D, renal function, and fibroblast growth factor (FGF)-23. Altered phosphate levels have been associated with reduced bone mineral density and increased skeletal fragility, highlighting its importance in maintaining bone integrity [9].

Understanding the relationship between systemic biochemical markers and RRR is important, particularly in the Indian population where nutritional deficiencies and metabolic bone disorders are common. Limited studies have correlated mandibular RRR with systemic biochemical parameters, especially using digital Wical and Swoope analysis. Therefore, the present study was conducted to evaluate mandibular RRR radiographically and assess its association with selected systemic biochemical markers in completely edentulous individuals. The secondary objectives were to assess gender-related differences in mandibular RRR and biochemical marker levels and to explore variables independently associated with mandibular RRR. The null hypothesis was that no correlation exists between vertical mandibular RRR and serum levels of vitamin K, vitamin D, calcium, phosphorus, or ALP in completely edentulous individuals.

## Materials and methods

Study design

This clinical, observational, cross-sectional study was conducted to evaluate mandibular RRR and its association with systemic biochemical markers, including vitamin K, vitamin D, calcium, phosphorus, and ALP.

Ethical considerations

Ethical approval was obtained from the Institutional Ethics Committee, Srirama Chandra Bhanja Dental College and Hospital, Cuttack, Odisha. The study adhered to the principles of the World Medical Association Declaration of Helsinki (1975, revised 2013). It was registered with the Clinical Trial Registry-India (CTRI No. REF/2025/12/099118). Written informed consent was obtained from all participants, and confidentiality was strictly maintained.

Study setting

The study was conducted at S.C.B. Dental College and Hospital, Cuttack, Odisha, involving the Departments of Prosthodontics (patient recruitment and clinical procedures) and Oral Medicine and Radiology (radiographic evaluation), along with the Department of Biochemistry at S.C.B. Medical College for serum biomarker analysis.

Study population and sample size

The sample size was calculated using an alpha error of 10%, a 90% confidence level, 80% statistical power, and the correlation coefficient reported in a previous study, with an adjustment for the anticipated non-response rate. Based on these parameters, the required sample size was determined to be 54 participants [10]. Accordingly, participants were selected from patients reporting to the Department of Prosthodontics and Crown & Bridge based on predefined inclusion and exclusion criteria (Table 1).

**Table 1:** Inclusion and exclusion criteria of the study participants

Category	Criterion
Inclusion criteria	Completely edentulous participants
	Age between 45 and 80 years
	Edentulous for more than one year
	Willing to participate and provide informed consent
Exclusion criteria	History of pre-prosthetic surgical procedures
	Congenital or acquired maxillofacial defects
	Neurological disorders affecting bone metabolism
	Metabolic bone diseases (e.g., osteoporosis, Paget's disease)
	Malignancy involving bone
	Chronic renal impairment
	Autoimmune disorders affecting bone metabolism
	Current or previous use of medications influencing bone metabolism (e.g., bisphosphonates, corticosteroids, hormone replacement therapy, calcium channel blockers)
	Current vitamin or mineral supplementation
	Previous denture use for more than two years

Radiographic evaluation

**Figure 1:**
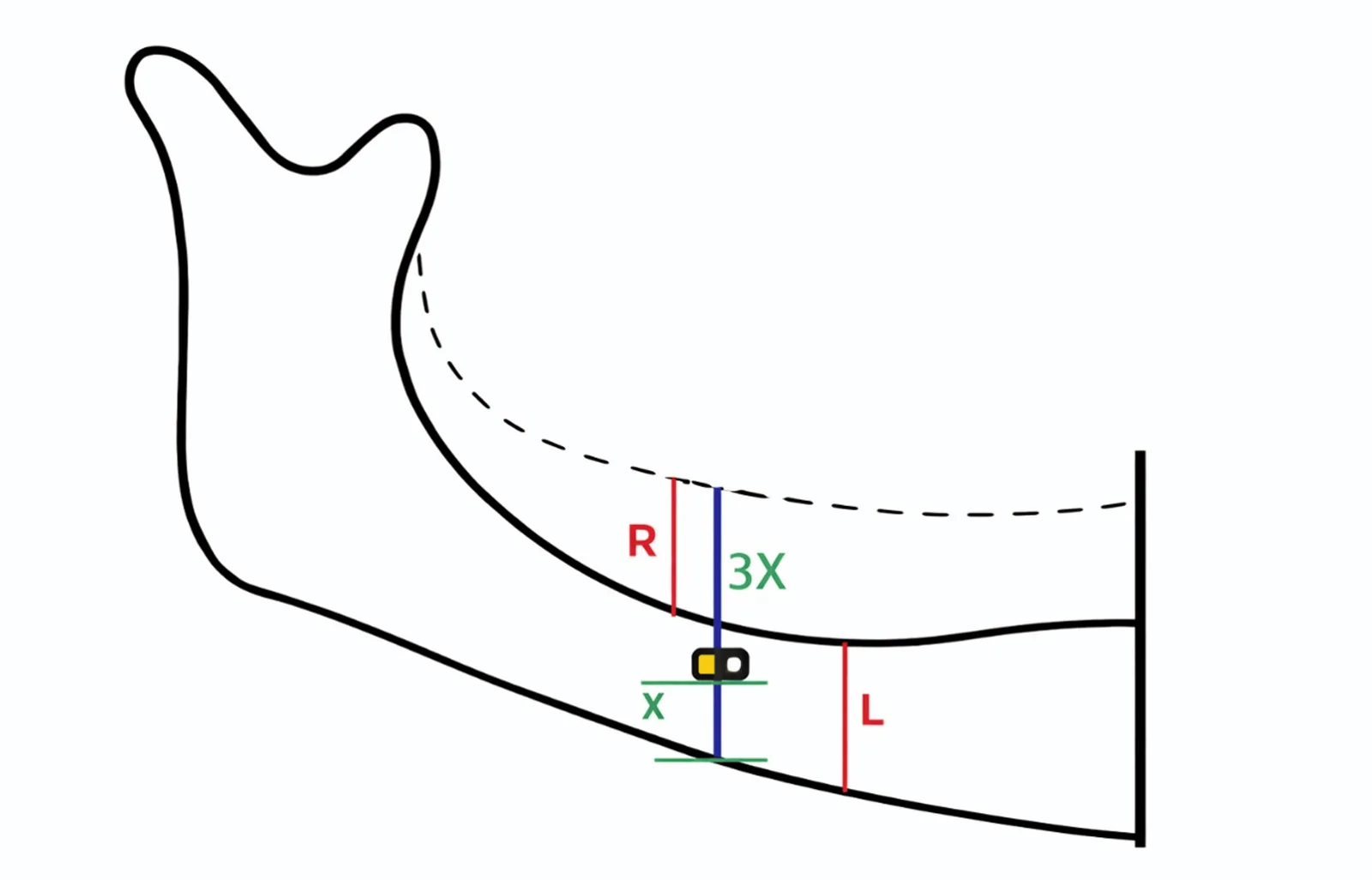
Schematic illustration of the Wical and Swoope method for estimating original mandibular height and assessing residual ridge resorption using the mental foramen as a reference landmark Image credit: Created by the authors and edited using ChatGPT (OpenAI, California, US).

Digital panoramic radiographs were obtained using a Vatech PaX-i system (Vatech Co., Ltd., Republic of Korea) at 73 kVp, 10 mA, and 13 seconds exposure time. Mandibular RRR was assessed using the Wical and Swoope method (Figure 1).

**Figure 2:**
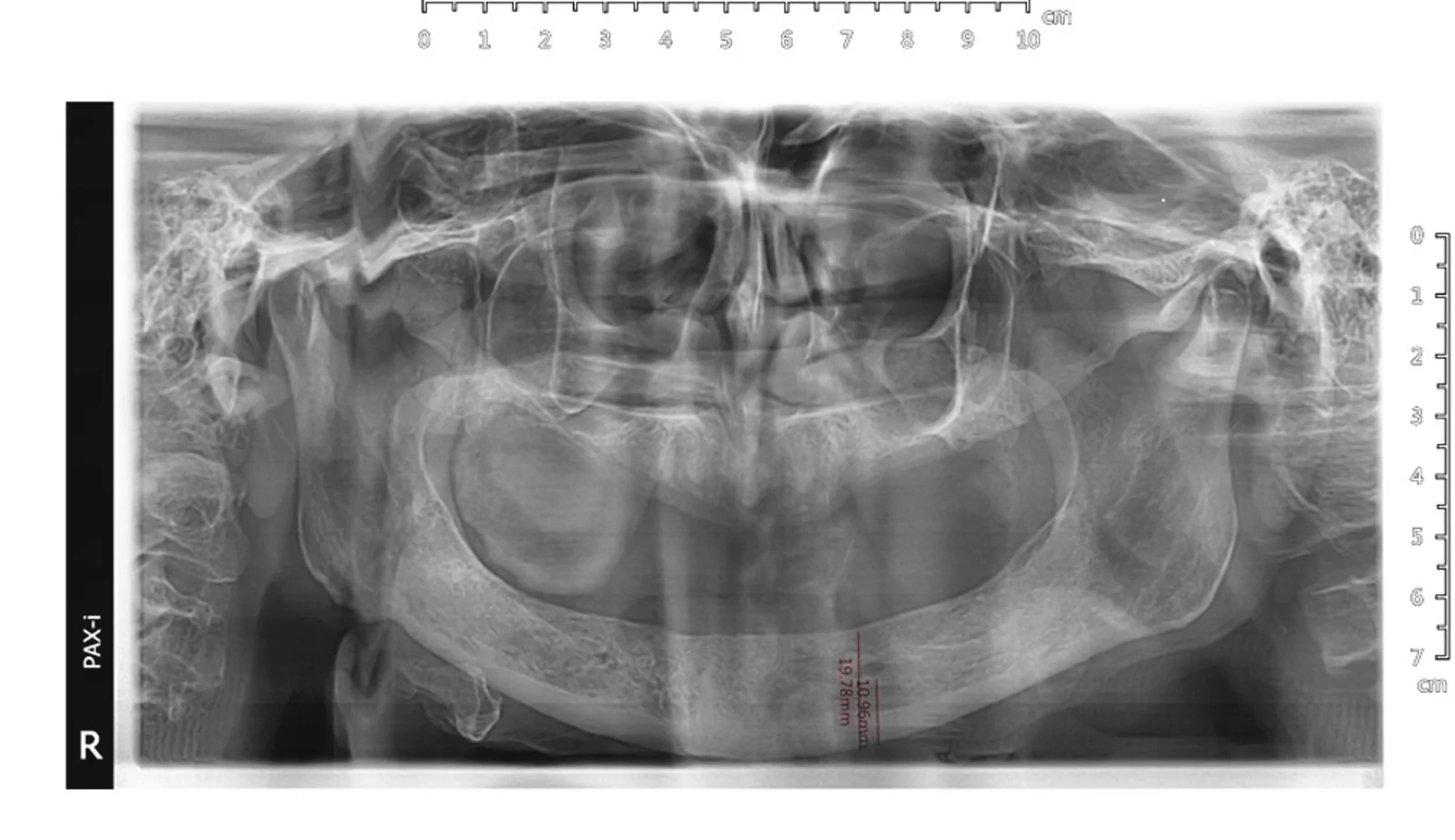
Orthopantomogram (OPG) showing linear measurement of mandibular residual ridge resorption

The distance from the inferior border of the mandible to the inferior border of the mental foramen (X) and the total mandibular height at the mental foramen region (L) were measured on the side exhibiting the clearest radiographic visibility of the mental foramen (Figure 2).

Biochemical analysis

Approximately 5 mL of venous blood was collected from the antecubital vein of each participant under aseptic conditions and transferred into plain and tripotassium ethylenediaminetetraacetic acid (K₃EDTA) vacutainer tubes. The samples were centrifuged at 3000 rpm for 10 minutes to separate the serum, which was subsequently stored under appropriate conditions until biochemical analysis.

**Figure 3:**
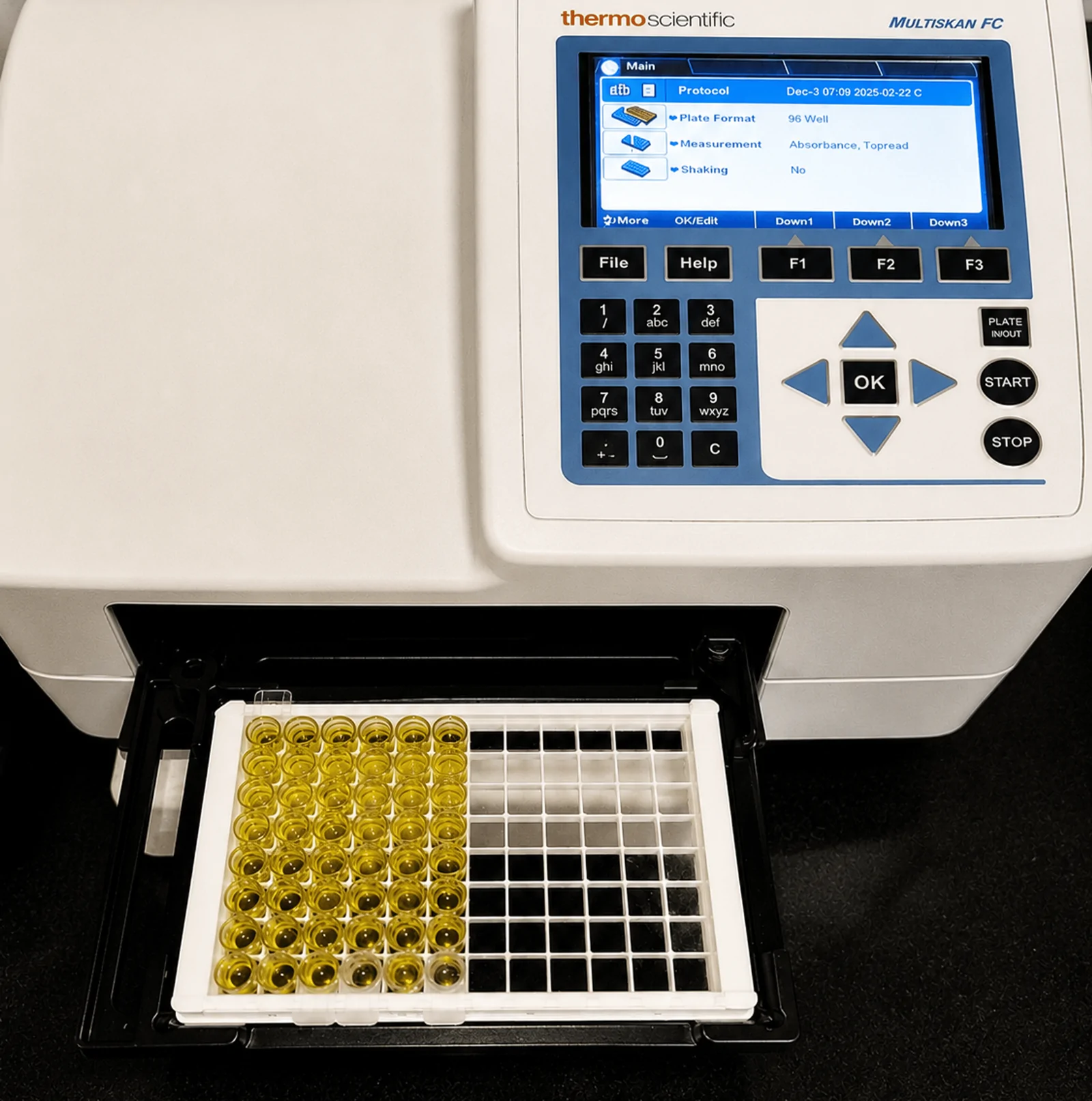
Thermo Scientific Multiskan™ FC microplate photometer used for measuring optical density during the enzyme-linked immunosorbent assay (ELISA)

Serum vitamin K1 (phylloquinone) levels were estimated using a commercially available enzyme-linked immunosorbent assay (ELISA) kit (Abbkine Inc., USA). The assay was based on a sandwich immunoassay principle, and vitamin K concentrations were determined from a standard calibration curve (Figure 3).

The results were expressed in ng/mL.

Serum 25-hydroxyvitamin D (25(OH)D) levels were measured using the Alinity i Immunoassay Analyzer (Abbott Diagnostics, USA) employing a chemiluminescent microparticle immunoassay (CMIA).

Serum ionized calcium levels were determined using an ion-selective electrode (ISE)-based electrolyte analyzer (HDC Lyte Pro, HDC Medica Corporation, India). This technique measures biologically active free calcium ions in serum through a calcium-selective membrane electrode, and the results were recorded in mmol/L.

Serum inorganic phosphorus levels were analyzed using the Alinity c Clinical Chemistry Analyzer (Abbott Diagnostics, USA) by the photometric phosphomolybdate method. Inorganic phosphate reacts with ammonium molybdate to form a phosphomolybdate complex, the absorbance of which is directly proportional to the phosphorus concentration.

Serum ALP activity was measured using the Alinity c Clinical Chemistry Analyzer (Abbott Diagnostics, USA) according to the International Federation of Clinical Chemistry (IFCC)-recommended kinetic method. The assay is based on the enzymatic hydrolysis of p-nitrophenyl phosphate to p-nitrophenol.

All biochemical analyses were performed in accordance with the manufacturers’ instructions under standardized laboratory conditions. Appropriate internal quality control procedures were implemented throughout the study to ensure the accuracy, reliability, and reproducibility of the obtained measurements.

Statistical analysis

The data were analysed using IBM SPSS Statistics for Windows, Version 25 (Released 2017; IBM Corp., Armonk, New York, United States). Correlation between mandibular RRR values and biochemical factors was assessed using Spearman correlation analysis. The Mann-Whitney U test was used to compare biochemical parameters between males and females. Stepwise linear regression analysis was performed to identify biochemical factors associated with mandibular RRR.

## Results

Demographics

**Figure 4:**
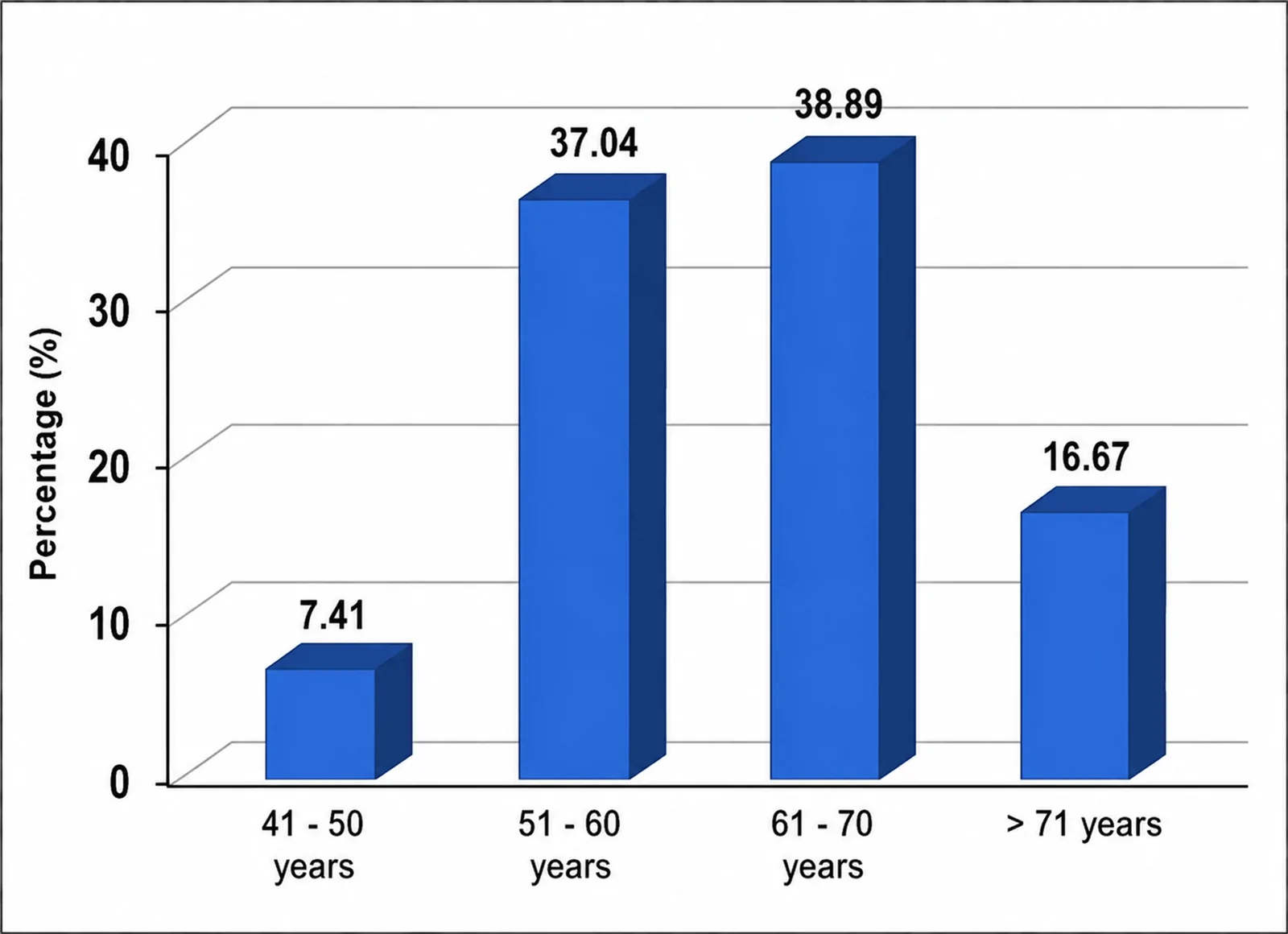
Distribution of study participants according to age group

A total of 54 participants were included. The majority of participants were aged 61-70 years (n=21, 38.9%), followed by 51-60 years (n=20, 37.0%), >70 years (n=9, 16.7%), and 41-50 years (n=4, 7.4%) (Figure 4).

**Figure 5:**
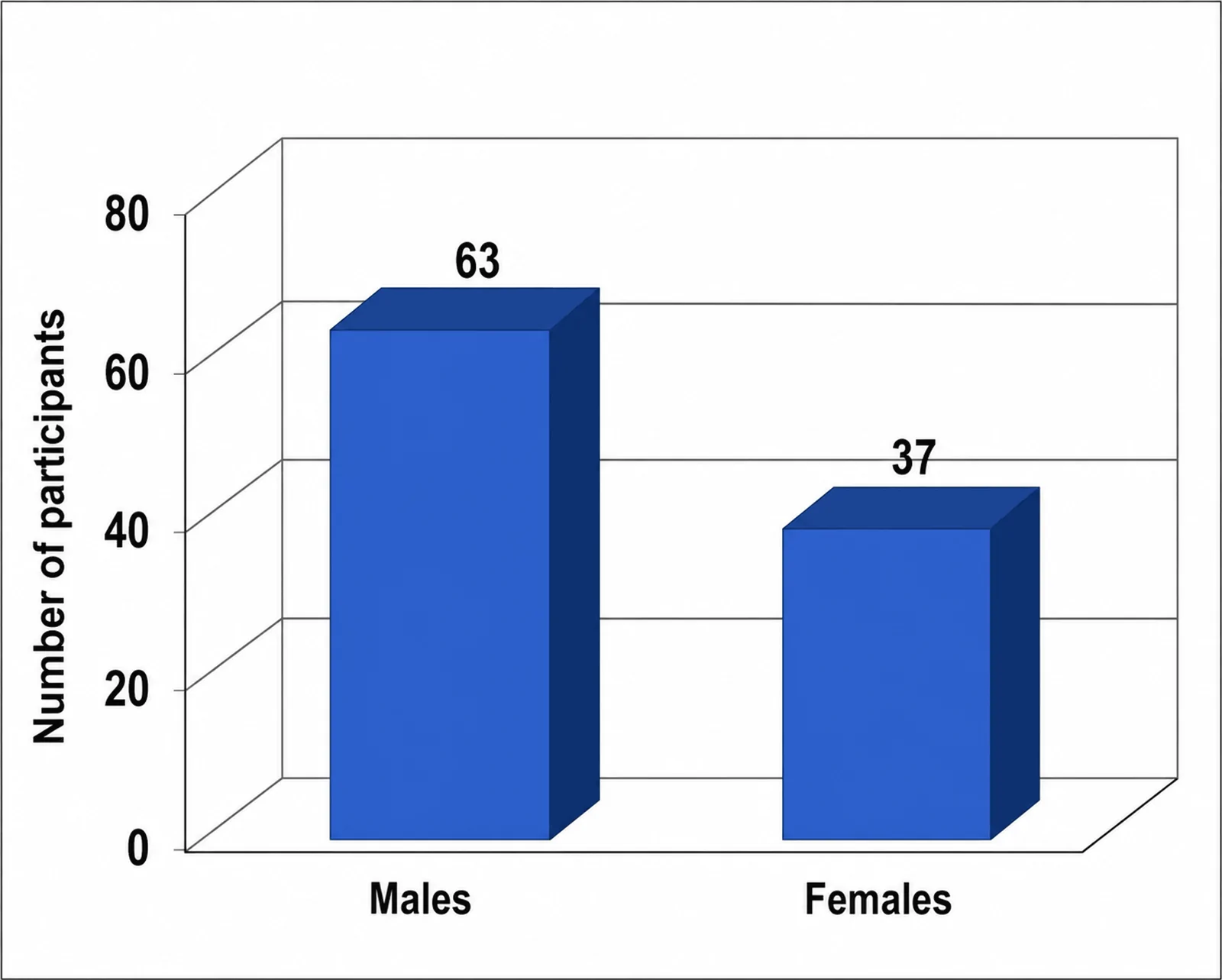
Distribution of study participants according to gender

Males constituted 63.0% of the study population, while females accounted for 37.0% (Figure 5).

Correlation analysis

**Figure 6:**
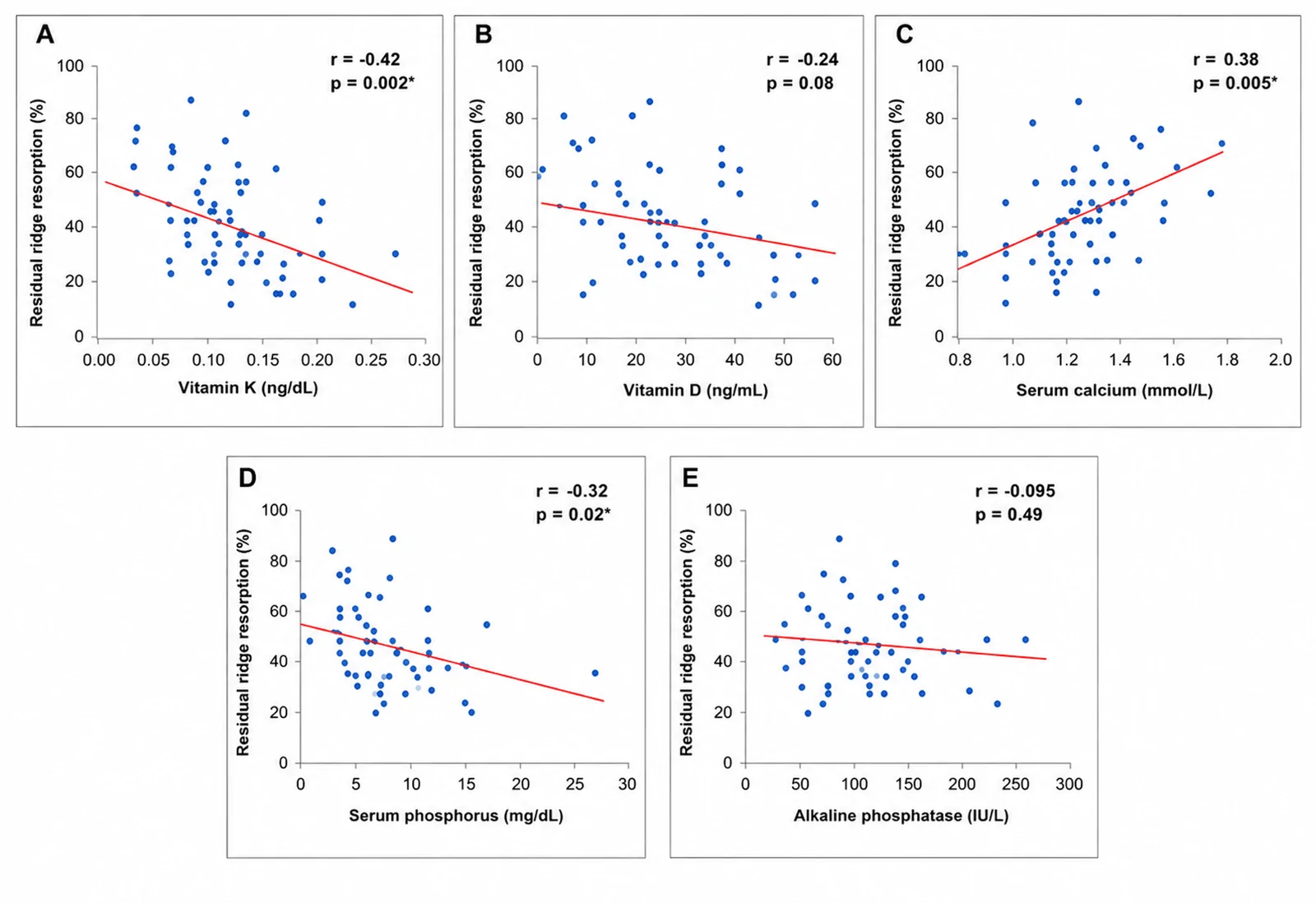
Scatterplots illustrating the correlation between mandibular residual ridge resorption and serum biochemical parameters (A) Serum vitamin K, (B) serum vitamin D, (C) serum calcium, (D) serum phosphorus, and (E) serum alkaline phosphatase. Each scatterplot shows the relationship between mandibular residual ridge resorption and the respective biochemical parameter. The red line represents the fitted linear regression. r denotes the Spearman correlation coefficient, and the corresponding p value is shown for each scatterplot. ^*^Statistically significant (p<0.05).

RRR showed a significant negative correlation with serum vitamin K (r=−0.42, p=0.002) and serum phosphorus (r=−0.32, p=0.02), and a significant positive correlation with serum calcium (r=0.38, p=0.005). No significant association was observed between RRR and vitamin D (r=−0.24, p=0.08), alkaline phosphatase (r=−0.095, p=0.49), or duration of edentulousness (r=−0.028, p=0.84). Low serum vitamin K levels were predominantly observed in males (77.27%) (Figure 6).

Gender-based comparison

Females exhibited a slightly longer duration of edentulousness, while males showed higher mean RRR; however, these differences were not statistically significant. No significant gender differences were observed in serum levels of vitamin K, vitamin D, calcium, phosphorus, or ALP (Table 2).

**Table 2:** Comparison of mean biochemical parameters between males and females SD: Standard deviation; NS: Not significant. Comparisons between males and females were performed using the Mann–Whitney U test. ^*^Statistically significant (p<0.05).

		Number	Mean	SD	mean rank	Z	p value
Vitamin K (ng/dL)	Males	30	0.11	0.04	29.04	-1	p = 0.31
	Females	24	0.12	0.044	24.8		NS
Vitamin D (ng/mL)	Males	30	20.6	10.1	29.2	-0.94	p = 0.34
	Females	24	17.9	7.6	24.5		NS
Serum calcium (mmol/L)	Males	30	1.17	0.11	24.8	-1.08	p = 0.27
	Females	24	1.15	0.12	31.9		NS
Serum phosphorus (ng/dL)	Males	30	7.4	5.8	25.4	-1.5	p = 0.1
	Females	24	9.19	6.7	31.05		NS
Serum alkaline phosphatases	Males	30	104	42.8	28.7	-1.2	p = 0.2
(IU/L)	Females	24	113.4	36.8	25.3		NS

Regression analysis

Stepwise linear regression identified vitamin K as a significant predictor of RRR in the overall sample (R²=0.142, p=0.003) and among males (R²=0.096, p = 0.04). In females, serum calcium emerged as a significant predictor (R²=0.253, p=0.014). An increase in vitamin K levels was associated with reduced RRR, whereas higher serum calcium levels were associated with increased resorption in females (Table 3).

**Table 3:** Stepwise linear regression analysis to predict ride resorption using biochemical factors for overall sample and for both genders Unstd. Coef.: Unstandardized coefficient; SE: Standard error; Adj. R²: Adjusted coefficient of determination. p<0.05 was considered statistically significant. ^*^Statistically significant for the predictor variable; ^**^Statistically significant for the regression model.

			Unstd. Coef.	SE	t	p value	Adj. R^2^	F	p value
Overall	Resorption	Constant	18.521	1.6	11.3	p=0.001^*^	0.142	9.74	p=0.003^**^
		Vitamin K	-40.28	12.9	-3.21	p=0.003^*^			
Males	Resorption	Constant	18.1	2.06	8.7	p=0.001^*^	0.096	4.48	p=0.04^**^
		Vitamin K	-35.48	16.7	-2.1	p=0.042^*^			
Females	Resorption	Constant	-10.17	8.6	-1.17	p=0.25	0.253	7.42	p=0.014^**^
		S. Calcium	20.35	7.4	2.72	p=0.014^*^			

## Discussion

The findings of the present study provide insight into the relationship between mandibular RRR and selected biochemical markers of bone metabolism, namely serum vitamin K, vitamin D, calcium, phosphorus, and ALP, as well as the influence of demographic factors such as age and gender in edentulous individuals.

Most participants were in the 61-70 years age group, followed by those aged 51-60 years. Although advancing age is associated with increased bone resorption and reduced bone formation, RRR is influenced by multiple factors, including nutritional, hormonal, and metabolic status. No significant gender-based differences in RRR were observed in the present study, consistent with the findings of Kalavathy et al. [10].

A significant negative correlation was observed between serum vitamin K levels and RRR, indicating that higher vitamin K levels were associated with reduced mandibular bone loss. Participants with vitamin K deficiency exhibited greater mean ridge resorption than those with normal levels. These findings support the role of vitamin K in bone metabolism through osteocalcin activation and enhanced bone mineralization, consistent with the observations of Bügel et al. [11].

Previous interventional studies have demonstrated the beneficial effects of vitamin K supplementation on bone health. Consistent with these findings, the present study revealed a significant association between baseline serum vitamin K levels and RRR [12]. Furthermore, regression analysis identified vitamin K as a significant predictor of RRR, particularly among males, suggesting a potential protective role in maintaining alveolar bone integrity. Serum vitamin D levels showed a weak negative correlation with RRR that was not statistically significant. Participants with vitamin D deficiency exhibited greater mean ridge resorption than those with normal levels, suggesting a potential role of vitamin D in mandibular bone health. The lack of significance may be attributable to variations in sunlight exposure, dietary habits, and lifestyle factors. Similar findings were reported by Chhantyal et al. [13]. Furthermore, Bolland et al. and Avenell et al. reported limited effects of vitamin D supplementation alone on bone mineral density and fracture prevention, supporting the present findings that vitamin D may exert only an indirect influence on RRR [14,15].

A significant positive correlation was observed between serum calcium levels and RRR. Elevated serum calcium may reflect increased skeletal resorption and release of calcium into the circulation rather than enhanced bone formation. Similar associations between higher serum calcium levels and reduced bone mineral density have been reported by López-Pérez et al. and Liu et al. [16,17]. Longitudinal studies have similarly reported greater declines in bone mineral density among individuals with higher baseline serum calcium levels [18]. Unlike previous studies assessing total serum calcium, the present study evaluated ionized calcium, the biologically active fraction, providing a more physiologically relevant measure of calcium status. Regression analysis identified serum calcium as a significant predictor of RRR among female participants, suggesting a greater influence of calcium metabolism on mandibular bone changes in women [19].

A weak but statistically significant negative correlation was observed between serum phosphorus levels and RRR, consistent with the findings of Kalavathy et al. [10]. Female participants showed higher mean serum phosphorus levels than males; however, the difference was not statistically significant. Similar findings have been reported in previous studies, which observed higher serum calcium and phosphate levels in women over 45 years of age compared to age-matched men [20].

No statistically significant association was observed between serum ALP levels and RRR. Although ALP is an established marker of bone formation, total serum ALP may not accurately reflect localized mandibular bone remodelling. The mean ALP levels in the present study were within the normal reference range, suggesting that routine ALP measurements may not detect subtle alterations in bone metabolism among apparently healthy individuals [21]. Furthermore, ALP reflects systemic bone metabolic activity, whereas mandibular ridge resorption is a localized multifactorial process. BALP is considered a more sensitive and specific marker of skeletal metabolism because it directly reflects osteoblastic activity [22]. Additionally, serum ALP levels may be influenced by several physiological and pathological conditions unrelated to mandibular bone resorption. Differences between the present findings and previous studies may also be explained by methodological variations, as most previous investigations evaluated bone metabolism using bone mineral density measurements, whereas the present study assessed RRR using linear radiographic measurements [23].

Females exhibited slightly higher mean levels of vitamin K, serum calcium, phosphorus, and ALP, while RRR was marginally greater in males; however, none of these differences were statistically significant. These findings suggest that factors other than gender may have a greater influence on mandibular RRR in the studied population [24].

Stepwise linear regression analysis identified vitamin K as the most significant predictor of RRR in the overall study population, accounting for approximately 14.2% of the variability in ridge resorption. Among male participants, vitamin K remained a significant predictor, whereas serum calcium emerged as a significant predictor among females. These findings suggest that different biochemical pathways may influence mandibular bone remodelling in males and females. Collectively, the results highlight the complex interplay between nutritional, metabolic, and systemic factors in the progression of RRR and emphasize the potential value of biochemical markers, particularly vitamin K and serum calcium, in understanding mandibular bone changes in edentulous individuals [8,25].

Limitations

This study has several limitations. The relatively small, single-center sample may limit the generalizability of the findings. Panoramic radiography, although widely used for residual ridge assessment, is less accurate than cone-beam computed tomography (CBCT) for three-dimensional evaluation. Formal assessment of radiographic measurement reliability was not performed, and only selected systemic biochemical markers were evaluated. In addition, potential confounding factors, such as smoking, diabetes mellitus, osteoporosis, and nutritional status, were not assessed and may have influenced the observed associations. Future multicenter longitudinal studies with larger sample sizes and a broader range of biochemical markers are warranted to validate these findings.

Clinical implications

The findings suggest that radiographic assessment of mandibular RRR, combined with evaluation of systemic biochemical markers, may provide valuable insight into bone metabolic status in edentulous individuals. Such an approach may facilitate risk assessment, improve treatment planning, and support strategies aimed at preserving alveolar bone and optimizing prosthodontic outcomes.

## Conclusions

Within the limitations of this study, systemic biochemical markers involved in bone metabolism were associated with mandibular RRR in completely edentulous individuals. Lower serum vitamin K levels were significantly associated with greater RRR, whereas serum calcium showed a significant positive association with mandibular bone loss. Serum phosphorus demonstrated a weak but significant inverse association with RRR, while serum vitamin D and ALP were not significantly associated with ridge resorption.
